# Testing Hypotheses on Risk Factors for Scientific Misconduct via Matched-Control Analysis of Papers Containing Problematic Image Duplications

**DOI:** 10.1007/s11948-018-0023-7

**Published:** 2018-02-19

**Authors:** Daniele Fanelli, Rodrigo Costas, Ferric C. Fang, Arturo Casadevall, Elisabeth M. Bik

**Affiliations:** 1 Department of Methodology, London School of Economics and Political Science, Columbia House, London, WC2A 2AE UK; 2 Centre for Science and Technology Studies (CWTS), Leiden University, P.O. Box 905, 2300 AX Leiden, The Netherlands; 3 Departments of Laboratory Medicine and Microbiology, University of Washington School of Medicine, Seattle, WA 98195 USA; 4 Department of Molecular Microbiology and Immunology, Johns Hopkins Bloomberg School of Public Health, Baltimore, MD 21205 USA; 5 uBiome, San Francisco, CA 94105 USA

**Keywords:** Scientific misconduct, Fraud, Fabrication, Falsification, Duplication, Research integrity, Gender, Pressures to publish

## Abstract

It is commonly hypothesized that scientists are more likely to engage in data falsification and fabrication when they are subject to pressures to publish, when they are not restrained by forms of social control, when they work in countries lacking policies to tackle scientific misconduct, and when they are male. Evidence to test these hypotheses, however, is inconclusive due to the difficulties of obtaining unbiased data. Here we report a pre-registered test of these four hypotheses, conducted on papers that were identified in a previous study as containing problematic image duplications through a systematic screening of the journal PLoS ONE. Image duplications were classified into three categories based on their complexity, with category 1 being most likely to reflect unintentional error and category 3 being most likely to reflect intentional fabrication. We tested multiple parameters connected to the hypotheses above with a matched-control paradigm, by collecting two controls for each paper containing duplications. Category 1 duplications were mostly not associated with any of the parameters tested, as was predicted based on the assumption that these duplications were mostly not due to misconduct. Categories 2 and 3, however, exhibited numerous statistically significant associations. Results of univariable and multivariable analyses support the hypotheses that academic culture, peer control, cash-based publication incentives and national misconduct policies might affect scientific integrity. No clear support was found for the “pressures to publish” hypothesis. Female authors were found to be equally likely to publish duplicated images compared to males. Country-level parameters generally exhibited stronger effects than individual-level parameters, because developing countries were significantly more likely to produce problematic image duplications. This suggests that promoting good research practices in all countries should be a priority for the international research integrity agenda.

**Electronic supplementary material:**

The online version of this article (10.1007/s11948-018-0023-7) contains supplementary material, which is available to authorized users.

## Introduction

The scientific literature is plagued by a small yet not negligible percentage of papers with fabricated or falsified results, the actual magnitude of which is unknown. Direct assessments in biomedical publications suggest that between 1 and 4% of papers contain problematic image manipulations, at least part of which appear to reflect intentional fabrication (Steneck 2006; Bik et al. 2016). More general data is offered by surveys, which suggest that 1–2% of scientists admit to having consciously fabricated or falsified data at least once (Fanelli 2009; John et al. 2012). Since respondents in these surveys are unlikely to have fabricated or falsified all of their publications, the actual proportion of the literature affected by misconduct or by a given form of questionable research practice is likely to be a fraction of the corresponding percentage of self-admission (Fiedler and Schwarz 2016).

Ultimately, all forms of survey data yield, by definition, only average estimates of the prevalence of misconduct, whereas the actual probability that a given scientific paper contains manipulated data is likely to vary depending on the research field, methodology, country and various other risk factors. The literature on research integrity offers multiple hypotheses about sociological, cultural and psychological factors that might increase the risk that scientists engage in misconduct. Testing these hypotheses is a matter of ongoing theoretical and empirical research. Particular attention has been paid to four major factors:

- *Pressures to publish* it is commonly suggested that scientists might engage in misconduct to keep up with high expectations of productivity and/or impact. This concern has already motivated numerous policies and initiatives aimed at discouraging scientists from publishing too much and/or from placing too high a value on journal impact factors (DFG 2010; VSNU 2015; DORA 2012). Pressures to publish may be higher and increasing in countries in which institutions are evaluated based on their publication performance (e.g. United Kingdom’s Research Excellence Framework), and/or in countries in which career advancement is determined by publications (e.g. tenure-track system in the United States of America) and/or in countries in which researchers are rewarded with cash for their high-impact publications [e.g. reward policies in China, see (Qiu 2010)]. The pressures to publish hypothesis is supported by perceptions and experiences reported in anonymous surveys (van Dalen and Henkens 2012; Anderson et al. 2007), but failed to predict the incidence of retractions and corrections (Fanelli et al. 2015), bias (Fanelli et al. 2017) or historical trends of scientists’ publication rate (Fanelli and Larivière 2016).
- *Social control* sociological and psychological theories suggest that individuals are less likely to engage in misconduct when scrutiny of their work is ensured by peers, mentors or society (Wright et al. 2008). An elaborate socio-economic analysis argued that the risk of scientific misconduct would be highest within “liberal research regimes adopted by developmental states and marked by freedom from government oversight, and illiberal laboratory cultures imported from Germany and marked by all-powerful lab directors and their vulnerable underlings” (Lee and Schrank 2010). The authors of this analysis predicted that mutual criticism and policing of misconduct might be least likely in developing countries in which academic institutions were built following the German model, and might be most likely in developed (i.e. highly regulated) countries with an Anglo-American (i.e. highly egalitarian) academic culture (Lee and Schrank 2010). This analysis listed examples of countries falling within each category as well as intermediate cases (Lee and Schrank 2010). Within teams, the social control hypothesis predicts that mutual criticism is likely to be directly proportional to the number of team members and inversely proportional to their geographic distance—a prediction supported by studies on retractions and bias in the literature (Fanelli et al. 2015; Fanelli 2012).
- *Misconduct policies* a growing number of countries and/or institutions are establishing official policies that regulate how suspected cases of misconduct can be identified, investigated and punished, following the rationale that clear rules and sanctions will have a deterrent effect (Resnik et al. 2015). Countries differ widely in how they define and enforce misconduct policies, and it is commonly suggested that the greatest deterrent effect would be obtained by misconduct policies that are legally enforceable [e.g. (Redman and Merz 2008)].
- *Gender* males are more prone to taking risks and are more status-oriented than females, and might therefore be more likely to engage in scientific misconduct (Fang et al. 2013). This hypothesis received some support by statistics about findings of misconduct by the US Office of Research Integrity (Fang et al. 2013). However, other interpretations of these data have been proposed (Kaatz et al. 2013), and gender did not significantly predict the likelihood to produce a retracted or corrected paper, once various confounders were adjusted for in a matched-control analysis (Fanelli et al. 2015).

Progress in assessing the validity of these hypotheses in explaining the prevalence of misconduct has been hampered by difficulties in obtaining reliable data. Survey results are very sensitive to methodological choices and, by definition, report what a sample of voluntary respondents think and are willing to declare in surveys—i.e. not necessarily what the average scientist actually thinks and does (Pupovac and Fanelli 2014; Fanelli 2009; Fiedler and Schwarz 2016). Retractions of scientific papers, most of which are due to scientific misconduct (Fang et al. 2012), offer a pool of actual cases whose analyses have yielded important insights (Grieneisen and Zhang 2012; Lu et al. 2013; Fang et al. 2012; Fanelli et al. 2015). Results obtained on retractions, however, may not be generalizable, because retractions still constitute a very small fraction of the literature and by definition are the result of a complex process that can be influenced by multiple contingent factors, such as level of scrutiny of a literature, presence of retraction policies in journals, and the scientific community’s willingness to act (Fanelli 2013).

An unprecedented opportunity to probe further into the nature of scientific misconduct is offered by a recent dataset of papers that contain image duplications of a questionable or manifestly fraudulent nature (Bik et al. 2016). These papers were identified by direct visual inspection of 20,621 papers that contained images of Western Blots, nucleic acid gels, flow cytometry plots, histopathology or other forms of image data. These papers had been published between the years 1995 and 2015 in 40 journals. Having been obtained by a systematic screening of the literature, this sample is free from most limitations and biases that affect survey and retraction data, and therefore offers a representative picture of errors and/or misconduct in the literature—at least with regard to image duplications in biomedical research. Descriptive analyses of these data have yielded new insights into the rate of scientific misconduct and its relative prevalence amongst different countries (Bik et al. 2016).

We conducted a pre-registered analysis (osf.io/w53yu) of data from Bik et al. (2016) to test, using a matched-control approach, multiple hypothesis about social and psychological risk factors for scientific misconduct. Our analysis focused on the largest and most homogeneous subsample of the original data set, i.e. 8138 papers published in the journal *PLoS ONE* between the years 2013 and 2014, the visual inspection of which led to identifying 346 papers with problematic image duplications.

Image duplications included in our sample could have been generated by unintentional error or intentional misconduct, and they were grouped according to their likelihood to result from the latter following a classification based on the level of duplication complexity (see (Bik et al. 2016) for further details):

- *Category 1* Simple duplications, in which the same image is presented twice to represent different conditions, possibly due to accidental mislabeling (N = 83).
- *Category 2* Duplications with re-positioning, in which one image has been shifted, rotated or reversed, suggesting some level of active intervention by the researcher (N = 186)
- *Category 3* Duplications with alteration, in which figures contained evidence of cutting, patching and other forms of substantive embellishment and manipulation which betrays a possible intention to mislead (N = 77).

Category 1 duplications are most likely due to error, whilst categories 2 and 3 are likely to contain a mixture of errors and intentional fabrications. Therefore, if factors predicted to affect scientific misconduct have any effect at all, such effects are predicted to be most relevant in categories 2 and 3 duplications and to have little or no effect on category 1 errors.

For each paper containing duplicated images we identified, from the remaining sample of N = 7792 screened papers, two controls that had been published in the same journal and time period, and that did not contain detectable signs of image duplication. We then measured a set of variables that were relevant to each tested hypothesis and run a conditional logistic regression analysis to test whether and how these variables were associated with the risk of committing scientific misconduct.

## Materials and Methods

Methods of this study followed very closely the protocol of a previous analysis of risk factors for retractions and corrections (Fanelli et al. 2015). To guarantee the confirmatory and unbiased nature of our analyses, all main and secondary analyses as well as sampling and analytical methodology were pre-specified and registered at the Center for Open Science (osf.io/w53yu) (Fanelli et al. 2016). The main goal of the study was to produce a matched-control retrospective analysis aimed at identifying which characteristics of paper and author were significantly predictive of the likelihood to fall into the “treatment” as opposed to “control” category (papers with or without problematic image duplications, respectively).

### Sampling of Papers

Papers containing image duplications had been identified and classified by the independent assessment of three authors (EB, AC, FF), in a previous publication (Bik et al. 2016). For each treatment paper, two controls were retrieved for inclusion. These control papers were retrieved from the set of papers that had also been screened by these authors and in which no evidence of data duplication had been found. The entire sample of papers (with or without duplications) was ordered by publication date, using the Web of Science unique identifier code as a proxy of publication order. Then, for each paper with image duplications, we selected the two closest control papers, i.e. one published immediately before and one immediately after the treatment paper. When candidate control papers overlapped, the next available control paper was selected instead. This procedure is a standard method to ensure that treatment and control papers are as similar as possible with regard to all confounding factors.

### Data Collection

Following previous protocols, we collected a set of relevant characteristics of all included papers and of all of their authors. In particular, for each paper we recorded:

- Number of authors of each paper.
- Number of countries listed in the authors’ addresses.
- Average distance between author addresses, expressed in thousands of kilometers. Geographic distance was calculated based on a geocoding of affiliations covered in the Web of Science (WOS) (Waltman et al. 2011).

Moreover, for each author of each paper we retrieved the following data from the Web of Science database:

- Year of first and last paper recorded in the WOS, as a proxy of earliest and latest publication.
- Total number of article, letter and review papers, as classified by the WOS.
- Total number of citations received by all papers from the author.
- Field-normalized citation score: a normalized score that for each author’s paper, aggregates citations received, adjusting for the WOS Subject Category of the paper’s journal and for year of publication.
- Field-normalized journal impact score: a normalized score that for each author’s paper, aggregates the impact factor of the paper’s journal, adjusting for average impact factor of journals within the corresponding WOS Subject Category and year.
- Proportion of papers published in the top-10 journals: Proportion of papers published in the top-10% most cited papers of that author’s fields.
- Author’s main country of activity, based on the address most commonly indicated as the author’s address within all of his/her publications.
- Author’s first name. The combination of first name and country was used to assign gender. The majority of gender assignments were made by a commercial service (genderapi.com) and subsequently completed by hand by one of the authors, who is not familiar with East-Asian names (DF). All main analyses reported in the text are based on this gender variable. A research assistant of East-Asian origin subsequently attempted to classify by hand the gender of all remaining unassigned names. All analyses were repeated using this latter gender variable and all results are reported in the Supplementary Information (variable indicated as “genderHand”). When an author’s gender could not be classified reliably by either method, gender was assigned to the “unknown” category.

Country information was used to assign each author to the corresponding country-level variable, using the following scheme:

- *Publication incentives policies* i.e. cash-incentives to individuals (CN, KR, TU); performance linked to individual’s career (DE, ES, USA); performance linked to institution’s funding (AU, BE, NZ, DK, IT, NO, UK), based on classifications in (Franzoni et al. 2011).
- *Social control hypothesis* developmental state—German academic model (CN, JP, KR); intermediate case (DE, SI, TW, ISR); regulatory state and Anglo-American academic culture (US, UK), based on the classification by Lee and Schrank (2010).
- *Misconduct policy* national and legally enforced (USA, DK, NO); national non-legally enforced (UK, SW, FI, NL, DE, AT, AU, JP, CN, KR, CR, TN, ZA); local (institutional) policies (ES, IL, FR, BE, CH, EE, LV, PL, CZ, HU, PE, GR, IN, BD), data based on references in (Fanelli et al. 2015).

Although we collected information for all authors of the papers, we only tested individual predictors measured on the first and last authors, positions that in biomedical papers tend to be attributed to the authors that contributed most to the research, often in the role of junior and senior author, respectively (Pontille 2004; Costas and Bordons 2011).

### Analyses

All variables were included in the analysis untransformed, although a few variables were re-scaled linearly. Author publication rates were divided by 10, geographic distances by 1000, countries-to-author ratios were multiplied by 100 and the number of authors and the number of countries in papers was rescaled around the mean. This re-scaling of variables served the purpose of improving the interpretability of effect sizes and their visibility in the figures and had no impact on the results (i.e. on the estimated effect sizes and their statistical significance).

All hypotheses were tested using conditional logistic regression analysis, the model best indicated for a matched control study design on large samples. Logistic regression is the standard regression analysis technique when response data is binary—a paper with or without duplicated images, in our case. Conditional logistic regression is simply a logistic regression that amongst its explanatory variables includes a dummy variable for each subgroup consisting of treatment + matched controls. Given *h* strata consisting of two controls and one paper with image duplications, and given *k* explanatory variables, our analysis calculated the log odds that a paper contains image duplications as:

1 $$\log \left( {\frac{p}{1 - p}} \right) = \alpha + \alpha_{2} s_{2} \ldots + \alpha_{h} s_{h} + \beta_{1} x_{1} + \cdots + \beta_{k} x_{k}$$

in which *s*_i_ is a dummy variable to represent stratum i. Equation 1 would be problematic to estimate with multiple strata, due to the large number of variables and the corresponding small number of residual degrees of freedom. However, the maximum likelihood estimation for conditional logistic regression avoids this problem by computing implicitly (“conditioning out”) the first h terms on the right-hand side of Eq. 1. Therefore, similarly to standard linear regression, results reported in the text and in the Supporting Information (SI) can be interpreted as the effect, on the (log) odds of containing image duplications, of increasing by one unit the value of a study or author characteristic, controlling for time and other differences between strata. By taking the exponential of these beta coefficients we obtain the corresponding odds ratio, which are used to present and interpret all data in graphs.

The conditional logistic regression approach is most useful when papers differ widely in important counfounding characteristics, such as year, month and journal of publication [see Fanelli et al. (2015)]. Our sample was homogeneous with regards to journal of publication and probably most other confounding variables. Therefore, analyses were also repeated with non-conditional logistic regression to assess the robustness of the results.

Following the suggestion of an anonymous peer-reviewer, we conducted additional robustness analyses using multilevel logistic regression, assuming random differences in the intercept of effects between countries. Since the study was not designed for this type of analysis, and many countries were under-represented, the maximum likelihood estimation (MLE) algorithm occasionally failed to converge. To facilitate convergence, all interval-scale variables were standardized by subtracting the mean and dividing by standard deviation, and between-country random effects were only assessed for countries with five or more events (paper with duplicated images) in our sample. For first authors, this entailed estimating random effects across the USA, Canada, France, India, Italy, China, South Korea, Taiwan, UK versus all other countries. For last authors, it entailed estimating random effects across the USA, Canada, India, Italy, China, South Korea, Taiwan, versus all other countries.

Analyses were conducted with all three categories of duplication combined, separately on categories 1, 2 and 3, and combining categories 2 and 3.

Since the sample size was pre-determined, we did not conduct a prospective power analysis. We conducted a post hoc power analysis based on unconditional logistic regression, which suggests that our main univariate analyses, when combining papers from all duplication categories (a total of 1044 data points) had over 99% statistical power to detect a small effect size (i.e. OR = 1.5), which was reduced to 89% for analyses restricted to the smallest subsample, i.e. category 3 duplications (a total of 237 data points). Regression analyses were conducted with the open-source statistical package Survival implemented by the statistical software R (Therneau 2014) and power analyses were implemented by the software G*Power v. Version 3.1.9.2 (Faul et al. 2009). The data set and code used to produce results and graphs were deposited on the same site in which the study was pre-registered (page: osf.io/9m8bs).

## Results

**Fig. 1:**
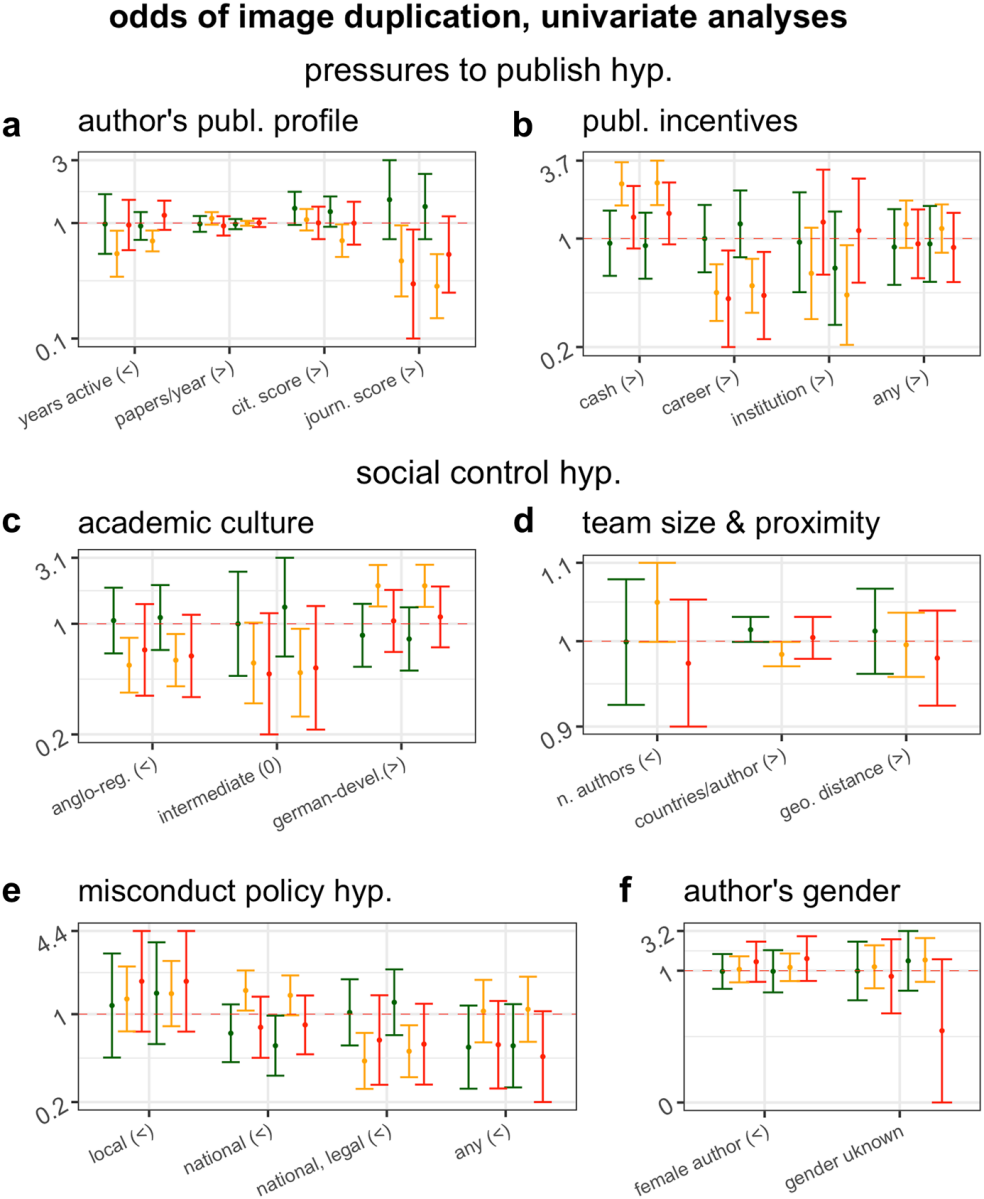
Effect (odds ratio and 95% CI) of characteristics of study and of first and last authors on the odds of publishing a paper containing a category 1 (green), category 2 (yellow) or category 3 (red) problematic image duplication. When six error bars are associated with one test, the first three error bars correspond to data from the first author and the last three are for data from the last author. Panels are subdivided according to overall hypothesis tested, and signs in parentheses indicate direction of expected effect (“>”: OR > 1; “<”: OR < 1; “0”: intermediate effect predicted). The more shifted the error bars are from the value of OR = 1 (dotted horizontal line), the larger the magnitude of effect measured. Bars that do not overlap with the OR = 1 line indicate an effect that is statistically significant at the 0.05 level or lower

Figure 1 reports the effects in each category of duplication of each tested parameter (i.e. odds ratio and 95% confidence interval), grouped by each composite hypothesis, with an indicated direction of the effect predicted by that hypothesis. In line with our overall predictions, category 1 duplications yielded a null association with nearly all of the parameters tested (Fig. 1, green error bars), and/or yielded markedly different effects from categories 2 and 3 papers (Fig. 1, orange and red bars, respectively). Sharp and highly significant differences between effects measured on the latter and the former duplication categories were observed for authors’ citation scores and journal scores (Fig. 1a), and for several country-level and team-level parameters (i.e. Figure 1b–e). No significant difference was observed amongst gender effects, which were all null (Fig. 1f).

Differences between effects measured on categories 2 and 3 duplications were not always consistent with our prediction that category 3 duplications should exhibit the largest effect sizes. For example, the number of years of activity of the author was only significantly associated with category 2 duplications (Fig. 1a). In most cases, however, the confidence intervals of effects measured for categories 2 and 3 were largely overlapping, suggesting that differences between categories 2 and 3 might be due to the smaller sample size (lower statistical power) achieved for the latter category. Overall, therefore, the results of univariable analyses were consistent with our predictions and with the original assessment of the status of these categories suggested by Bik et al. (2016): category 1 duplications are most likely to reflect genuine errors whilst categories 2 and 3 errors are most likely to reflect intentional manipulations. Hypotheses about determinants of scientific misconduct, therefore, are most directly testable on the latter two categories, which were combined in all subsequent analyses reported in the main text. All numerical results of all analyses reported in the main text as well as all secondary and robustness analyses obtained on each separate duplication category and on all categories combined are available as Supporting Information (data and code are available at osf.io/9m8bs).

**Fig. 2:**
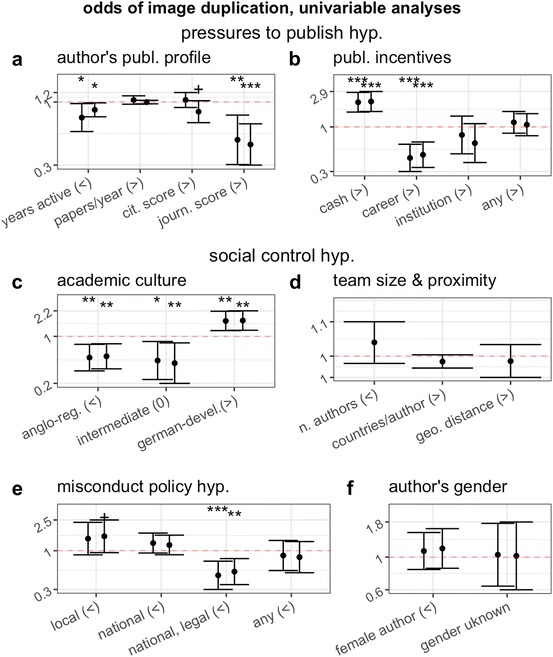
Effect (odds ratio and 95% CI) of characteristics of study and of first and last authors on the odds of publishing a paper containing a category 2 or 3 problematic image duplication. For each individual-level parameter, first and second error bars correspond to data from first and last authors, respectively. Panels are subdivided according to overall hypothesis tested, and signs in parentheses indicate the direction of expected effect (“>”: OR > 1; “<”: OR < 1; “0”: intermediate effect predicted). The more shifted the error bars are from the value of OR = 1 (dotted horizontal line), the larger the magnitude of the effect measured. Bars that do not overlap with the OR = 1 line indicate an effect that is statistically significant at the 0.05 level or lower. Conventional thresholds of statistical significance are flagged above each error bar to facilitate effect estimation (^+^*P* < 0.1; *P < 0.05; **P < 0.01; ***P < 0.001)

Results of univariable tests combining categories 2 and 3 papers together are in good agreement with the social control hypothesis (Fig. 2c) and partial agreement with the misconduct policy hypothesis (Fig. 2e). The gender hypothesis was not supported (Fig. 2f). The pressures to publish hypothesis was not or negatively supported by most analyses. In partial agreement with predictions, the risk of misconduct was higher in countries in which publications are rewarded by cash incentives (Fig. 2b) and was lower for researchers with a shorter publication time-span (i.e. presumably early-career researchers, Fig. 2a). Contrary to predictions, however, the risk of misconduct was lower for authors with a higher journal score (Fig. 1a) and in countries with publication incentive policies that are career-based and institutional-based, despite the case that the latter are those where pressures to publish are said to be highest (van Dalen and Henkens 2012).

**Fig. 3:**
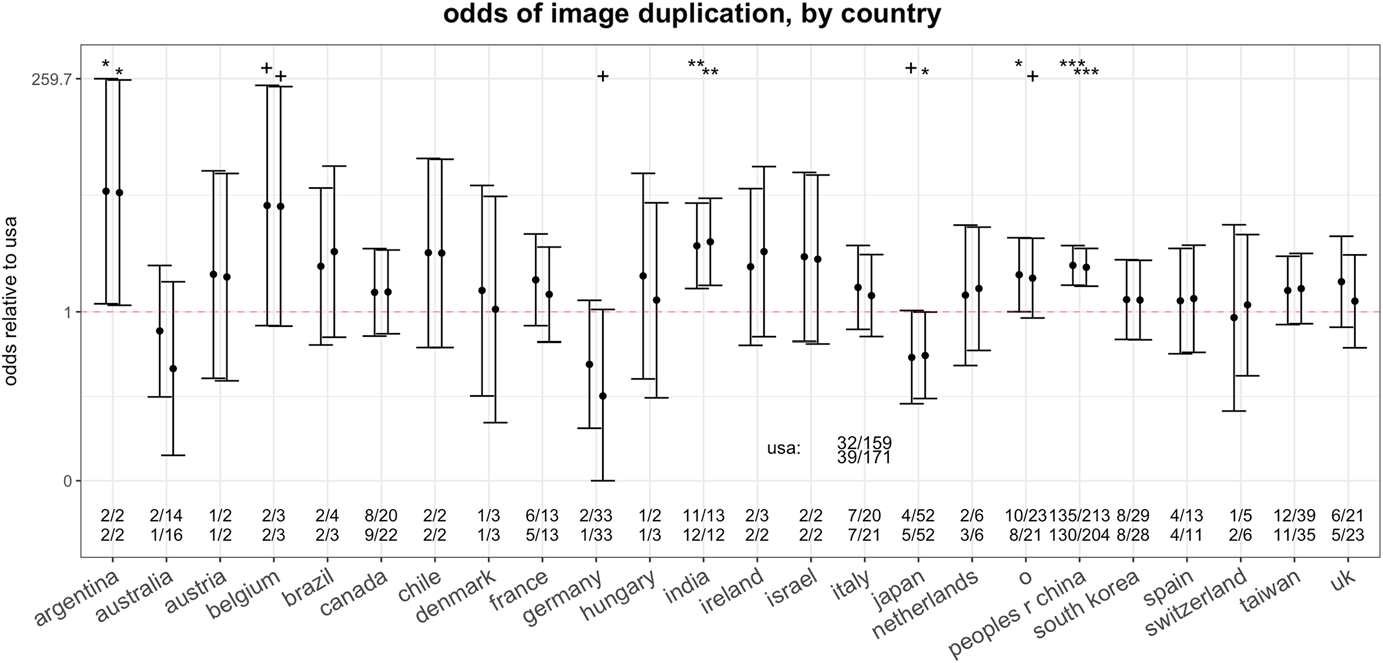
Effect (odds ratio and 95% CI) of country of activity of first and last authors on the odds of publishing a paper containing a category 2 or 3 problematic image duplication, compared to the United States. The data were produced with a multivariable model, in which dummy variables are attributed to countries that were associated with the first or last author of at least one treatment and one control paper. All other countries were included in the “other” category. Numeric data are raw numbers of treatment and control papers for first and last author (upper and lower row, respectively). The more shifted the error bars are from the value of OR = 1 (dotted horizontal line), the larger the magnitude of effect measured. Bars that do not overlap with the OR = 1 line indicate an effect that is statistically significant at the 0.05 level or lower. Conventional thresholds of statistical significance are flagged above each error bar to facilitate effect estimation (^+^*P* < 0.1; *P < 0.05; **P < 0.01; ***P < 0.001). The *P* values calculated for India and China (*P* = 0.002 and *P* < 0.001, respectively) remained significant at the 0.05 level if Bonferroni-corrected (i.e. divided by the total number of comparisons made, n = 24)

Overall, country-level parameters produced effects of larger magnitude (Fig. 2). Indeed, we observed sharp between-countries differences in the prevalence of image duplication (Fig. 3). Compared to the reference rate measured for the United States, image duplication was significantly higher amongst papers by authors from China, India, Argentina and other countries (i.e. those included in the “other” category, which are mostly developing countries, Fig. 3). Multiple other countries (e.g. Belgium, Austria, Brazil, Israel, etc.) also appeared to have higher risk than the United States but the small number of studies from these countries hampered the statistical power and thus our ability to draw any conclusion. Germany and Australia tended to have a lower risk than the United States, but only Japan had a statistically significant lower risk (Fig. 3).

These country estimates may incur in Type I error due to the multiplicity of tests involved (i.e. of countries being examined). However, even if we adopted a maximally conservative Bonferroni correction, and divided the measured z-scores (*P* values) by the total number of tests (n = 24), India and China would still emerge as having a significantly higher risk of data duplication at the 0.05 level (see legend in Fig. 3). The effects measured on these countries were large by conventional standards. The odds of containing a category 2 or 3 duplicated image for papers with a first or last author from India were 4.83 and 5.31 times the odds for papers with authors from the US (whose odds were low, i.e. 0.2 and 0.23, respectively). For authors from China, the corresponding effects were 3.03 and 4.62, and for other developing countries 2.42 and 2.23 (Fig. 3, see SI for all Odds Ratio ad 95% CI values). To put this result in perspective, if we hypothesized that the true probability of image duplication amongst US first authors is 1%, the corresponding estimated probability for researchers from India, China and other developing countries would be, respectively, 4.9, 3.1 and 2.4%.

To reduce the possible confounding effect of country in testing individual-level parameters, we performed secondary analyses on subsamples of countries with relatively homogeneous cultural and economics characteristics (Fig S1). Such sub-setting appeared to improve the detection of individual-level variables. In particular, the risk of duplication appeared to be positively associated with authors’ publication rate, citation score, journal score and female gender (Fig S1 a-h, and see SM for all numerical results). These effects, however, were never detected at conventional levels of statistical significance.

**Fig. 4:**
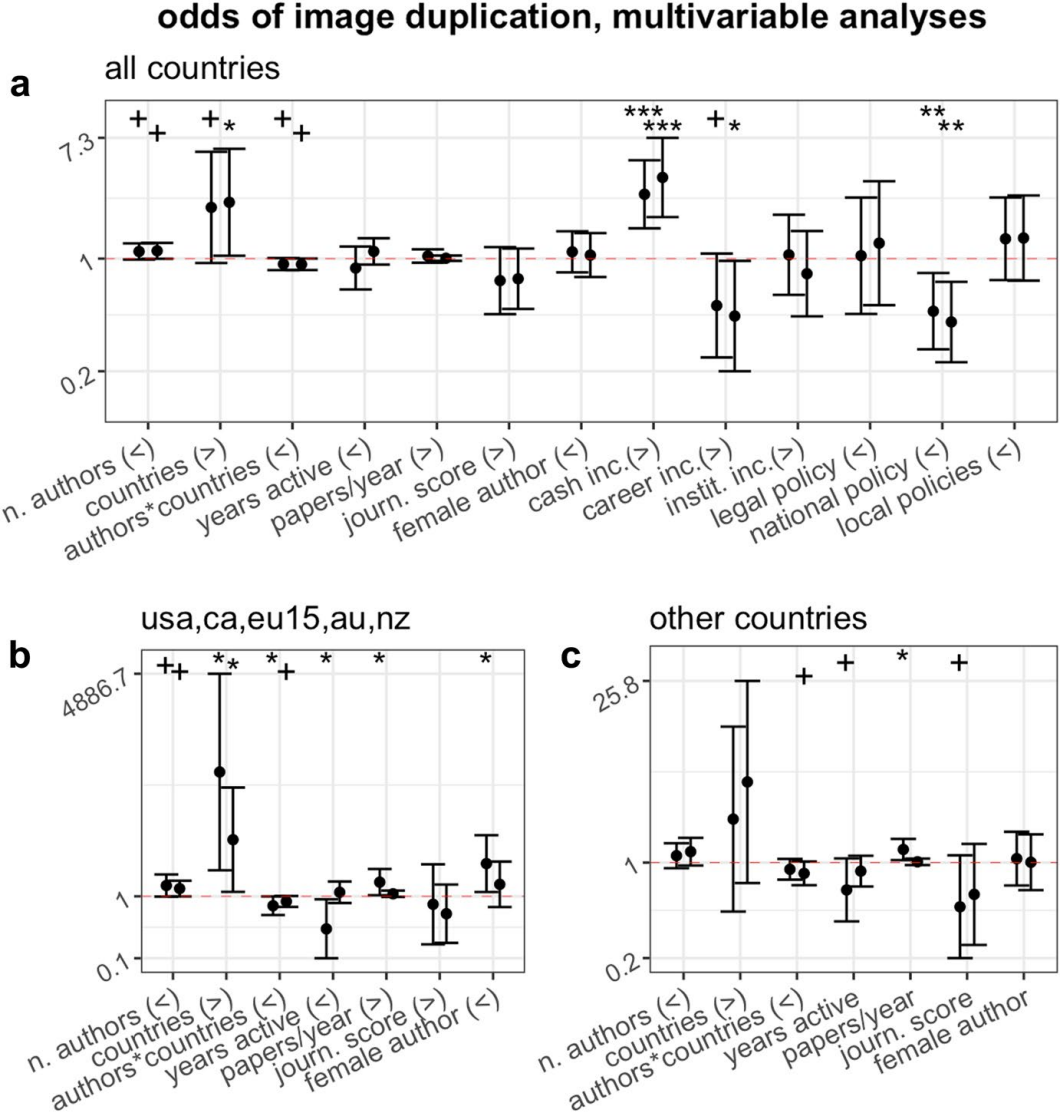
Effect (odds ratio and 95% CI) of characteristics of study and first and last author on the probability of publishing a paper containing a category 2 or 3 problematic image duplication. Each subpanel illustrates results of a multivariable model, partitioned by country subsets (see text for further details). First and second error bars correspond to data from first and last authors, respectively. Signs in parentheses indicate direction of expected effect (“>”: OR > 1; “<”: OR < 1). The more shifted the error bars are from the value of OR = 1 (dotted horizontal line), the larger the magnitude of effect measured. Bars that do not overlap with the OR = 1 line indicate an effect that is statistically significant at the 0.05 level or lower. Conventional thresholds of statistical significance are flagged above each error bar to facilitate effect estimation (^+^*P* < 0.1; *P < 0.05; **P < 0.01; ***P < 0.001)

Secondary multivariable analyses corroborated all of our main results (Fig. 4). A model that included individual parameters, as well as an interaction term between the number of authors and the number of countries (in place of the country-to-author ratio, which is not independent from the number of authors) and country-level parameters of publication and misconduct policies suggested that the risk of misconduct was predominantly predicted by country and team characteristics (Fig. 4a). The risk was significantly higher in countries with cash-based publication incentives, lower in those with national misconduct policies, and grew with team size as well as with number of authors, with the latter two factors modulating each other: for a given distance, larger teams were less at risk from misconduct, as the social control hypothesis predicted (Fig. 4a).

When limited to English-speaking and EU15 countries, multivariable analyses on first authors supported most theoretical predictions, suggesting that misconduct was more likely in long-distance collaborations and amongst early-career, highly productive and high-impact first authors (Fig. 4b). Female first authors were significantly more at risk of being associated with categories 2 and 3 duplications, a finding that is inconsistent with the gender hypothesis. Analyses on the remaining subset of countries yielded similar results (Fig. 4c).

Almost identical results were obtained with a non-conditional logistic regression model, consistent with the case that our sample was homogeneous with regards to important characteristics such as journal, methodology and year of publication. Secondary robustness analyses assuming between-country random effects also confirmed our main conclusions, by showing that most individual-level variables did not predict data duplication whereas most country-level analyses did. Indeed, these analyses measured significant or marginally non-significant effects for all incentive and misconduct policy categories. Results obtained combining all three categories of duplications were largely overlapping with those presented in the main text and would have led to similar conclusions (see all numerical results in SI).

## Discussion

To the best of our knowledge, this is the first direct test of hypotheses about the causes of scientific misconduct to be conducted on an unbiased sample of papers containing flawed or fabricated data. Our sample included papers containing honest errors and intentional fabrications in unknown relative proportions. However, we correctly predicted that category 1 duplications would exhibit smaller or null effects, whilst most significant effects, if observed at all, would be observed in categories 2 and 3 (Fig. 1, see pre-registration at osf.io/w53yu). Support of this prediction retrospectively confirms that, as suggested in a previous description of this sample (Bik et al. 2016), category 1 duplications are most likely the result of unintentional errors or flawed methodologies, whilst categories 2 and 3 duplications are likely to contain a substantial proportion of intentional fabrications.

Results obtained on categories 2 and 3 papers, corroborated by multiple secondary analyses (see SI), supported some predictions of the hypotheses tested, but did not support or openly contradicted other preditions:

- *Pressure to publish hypothesis* was either not supported, or partially supported. On the one hand, early-career researchers, and researchers working in countries where publications are rewarded with cash incentives were at higher risk of image duplication, as predicted. The correlation with cash incentives may not be taken to imply that such incentives were directly involved in the problematic image duplications, but simply that such incentives may reflect the value system in certain research communities that might incentivize negligence and/or misconduct in research.
- On the other hand, however, countries having other publication incentive policies had a null or even negative risk (Fig. 1b). In further refutation of predictions, an author’s individual publication rate and impact were not or negatively associated with image duplication. Surprisingly, in secondary multivariable analyses we observed a positive association between the publication rate of first authors and the risk of duplication (Fig S1). The latter finding might represent the first direct support of this prediction, or might be a false positive resulting from the multiple analyses conducted in this study. Future studies should be directed at confirming this result by repeating our protocol on a targeted sample of studies with image duplications or other proxies of scientific misconduct, published in the United States, EU15, Canada, New Zealand or Australia.
- *Social control hypothesis* was supported. In univariable analyses, predictions based on socio-cultural academic environments of different countries were in large agreement with observations (Fig. 1c). In multivariable analyses that adjusted for country characteristics, we observed a consistent negative interaction between the number of authors and the number of countries per author in a paper, which is in agreement with predictions stemming from this hypothesis (Fig. 4).
- *Misconduct policy hypothesis* was partially supported. Countries with national and legally enforceable policies against scientific misconduct were significantly less likely to produce image duplications (Figs. 1e, 4a). However, other misconduct policy categories were not associated with a reduced risk of image duplication, and tended if anything to have a higher risk. As already noted above with regard to the effect of publication incentive policies, this study cannot prove a cause-effect relationship. The negative association between the risk of image duplication and the presence of national misconduct policies may be explained by hypothesizing that scientists in countries with such policies follow higher standards of research methodology and integrity.
- *Gender hypothesis* was not supported. In none of the main and secondary analyses did we observe the predicted higher risk for males. Indeed, contrary to predictions, in secondary analyses we observed a significant positive association between female gender and risk of image duplication (Fig. 4b). Given the multiplicity of analyses conducted in this study, however, this latter finding may be spurious and should be corroborated by future confirmatory studies.

A previous, analogous analysis conducted on retracted and corrected papers and co-authored by two of us (DF and RC) had led to largely similar conclusions (Fanelli et al. 2015). The likelihood to correct papers for unintentional errors was not associated with most parameters, similarly to what this study observed for category 1 duplications. The likelihood to retract papers, instead, was found to be significantly associated with misconduct policies, academic culture, as well as early-career status and average impact score of first or last author, similarly to what was found here for categories 2 and 3 duplications.

Differently from what we observed on image duplications, however, the individual publication rate had been found in a previous analysis to be negatively associated with the risk of retraction and positively with that of corrections (Fanelli et al. 2015). We hypothesize that at least two factors may explain this difference in results. First, the previous study included retractions for every possible error and form of misconduct, including plagiarism, whereas the present analysis is dedicated to a specific form of error or manipulation. Second, analyses on retractions are intrinsically biased and subject to many confounding factors, because retractions are the end result of a complex chain of events (e.g. a reader signals a possible problem to the journal, the journal editor contacts the author, the author’s institution starts an investigation, etc.) which can be subjected to many sources of noise and bias. Results of this study, therefore, if on the one hand may be less generalizable to other forms of research misconduct, on the other hand are likely to be more accurate than results obtained on retractions.

 Results of this study are also in remarkable agreement with results of a recent assessment of the causes of bias in science, co-authored by two of us (Fanelli et al. 2017). This latter study tested similar hypotheses using identical independent variables on a completely different outcome (i.e. the likelihood to over-estimate results in meta-analysis) and using a completely different study design. This convergence of results is striking and strongly suggests that our quantitative analyses are detecting genuine underlying patterns that reflect a connection between research integrity and characteristics of the authors, team and country.

The present study has avoided many of the confounding factors that limit studies on retractions, but could not avoid other limitations. An overall limitation is that the kind of image duplication analyzed in this study is only one amongst many possible forms of data falsification and fabrication. This restriction limits in principle broad generalizations. However, as discussed above, our results are in large agreement with previous analyses that encompassed all forms of bias and/or misconduct (Fanelli et al. 2017; Fanelli et al. 2015), which suggests that our findings are consistent with general patterns linked to these phenomena.

Two other possible limitations of our study design make our results conservative. Firstly, we cannot ascertain which of the duplications were actually due to scientific misconduct and which to honest error, systematic error or negligence. Secondly, our individual-level analyses focused on characteristics of the first and the last author, under the assumption that these authors are most likely to be responsible for any flaws in a publication, but we do not actually know who, amongst the co-authors of each paper, was actually responsible. Both of these limitations ought to increase confidence in our results, because they are likely to have reduced the magnitude of measurable effect sizes. As our univariable analyses confirmed, image duplications that are most likely not due to scientific misconduct but to unintentional error are also less likely to be associated with any tested factor (Fig. 1). Similarly, if and to any extent that duplicated images had not been produced by first or last authors, then characteristics of first and last author should not be associated with the incidence of data duplication. Therefore, to any extent that our study was affected by either of these two limitations, these have simply introduced random noise in our data, reducing the magnitude of any measurable effect and thus making our results more conservative.

Based on conventions adopted in biomedical research, first authors are usually the team members who were mostly responsible for producing the data. This would suggest that first authors are also more likely to be responsible for data duplications compared to last authors, and therefore might exhibit stronger effects in the predicted direction. Across our analyses, we found very little differences between effects estimated on first and last authors. However, country was likely to be a significant confounding factor. In 93.8% of papers in our sample, first and last author were from the same country, which suggests that the characteristics of first and last authors were more similar within papers than between papers. Intriguingly, in secondary multivariable analyses restricted to a relatively homogenous subgroup of industrialized countries (USA, Canada, EU15, Australia and New Zealand), we observed that for first authors, all effects were observed to statistically significant levels, and with magnitudes significantly above those of last authors (Fig. 4b). This suggests again that, when significant confounding factors are taken into account, individual characteristics might indeed successfully predict the risk of scientific misconduct, and that first authors are the category most at risk. These results cannot be considered conclusive, due to their exploratory nature. Future studies should be designed to confirm these observations, which might be of exceptional importance to understand and predict research misconduct.

The possible presence of random error and noise in our data might have reduced the statistical power of our analyses, for the same reasons discussed above. However, our statistical power was relatively large. Even when restricted to the smallest subset (e.g. category 3 duplications) our analyses had over 89% power to detect an effect of small magnitude. We can therefore conclude that, despite the limitations discussed above, all of our tests had sufficient power to detect small effects and a high power to detect large and medium effects.

A further possible limitation in our analysis pertains to the accuracy with which we could measure individual-level parameters. Our ability to correctly classify the gender and to reconstruct the publication profile of each author was subject to standard disambiguation errors (Caron and Van Eck 2014) which may be higher for authors in certain subsets of countries. In particular, authors from South- and East-Asian countries have names that are difficult to classify, and often publish in local journals that are not indexed in the Web of Science and were therefore not captured by our algorithms. Any systematic bias or error in quantifying parameters for authors from these countries would significantly skew our results because country-level factors were found in this study, as well in previous studies on retractions, to have significant effects (Fanelli et al. 2015). However, we based all of our main conclusions on effects that were measured consistently in subsets of countries at lower risk of disambiguation error. Moreover, this limitation is only likely to affect the subset of tests that focused on author characteristics.

Indeed, one of the most important findings of this study is that significant individual-level effects might not be detectable unless country-level effects are removed or adjusted for, because the latter effects are prominent. We found strong evidence that problematic image duplications are overwhelmingly more likely to come from China, India and possibly other developing countries, consistent with the findings of a previous descriptive analysis of this data (Bik et al. 2016). Regardless of whether the image duplications that we have examined in this study were due to misconduct or unintentional error, our results suggest that particular efforts ought to be devoted to improving the quality and integrity of research in these countries.

In conclusion, we stress again that the present analysis of image duplications as well as previous analyses on retractions, corrections and bias (Fanelli et al. 2015, 2017) cannot demonstrate causality. However, all these analyses consistently suggest that developing national misconduct policies and fostering an academic culture of mutual criticism and research integrity might be effective preventive measures to ensure the integrity of future research in all countries.

## Electronic supplementary material

Below is the link to the electronic supplementary material.

Fig. S1 Effect (Odds Ratio and 95% CI) of characteristics of first and last author on probability of publishing a paper containing a category 2 or 3 image duplication. Each subpanel shows results of univariable analyses on subsets of countries (see text for further details). First and second error bar correspond to data from first and last authors, respectively. Panels are subdivided according to overall hypothesis tested, and signs in parentheses indicate direction of expected effect (“>” : OR > 1; “<” : OR < 1). The more shifted the error bars are from the value of OR = 1 (dotted horizontal line), the larger the magnitude of effect measured. Bars that do not overlap with the OR = 1 line indicate an effect that is statistically significant at the 0.05 level or lower. Conventional thresholds of statistical significance are flagged above each error bar to facilitate effect estimation (“+”: p < 0.1; “*”: P < 0.05; “**”: P < 0.01; “***”: P < 0.001) (TIFF 7264 kb)

Supplementary material 2 (TXT 242 kb)
