# Supplementary figures and images for: Testing Hypotheses on Risk Factors for Scientific Misconduct via Matched-Control Analysis of Papers Containing Problematic Image Duplications
Source: Sci Eng Ethics. 2018 Feb 19;25(3):771–89. doi: 10.1007/s11948-018-0023-7 (PMC6591179; doi:10.1007/s11948-018-0023-7)

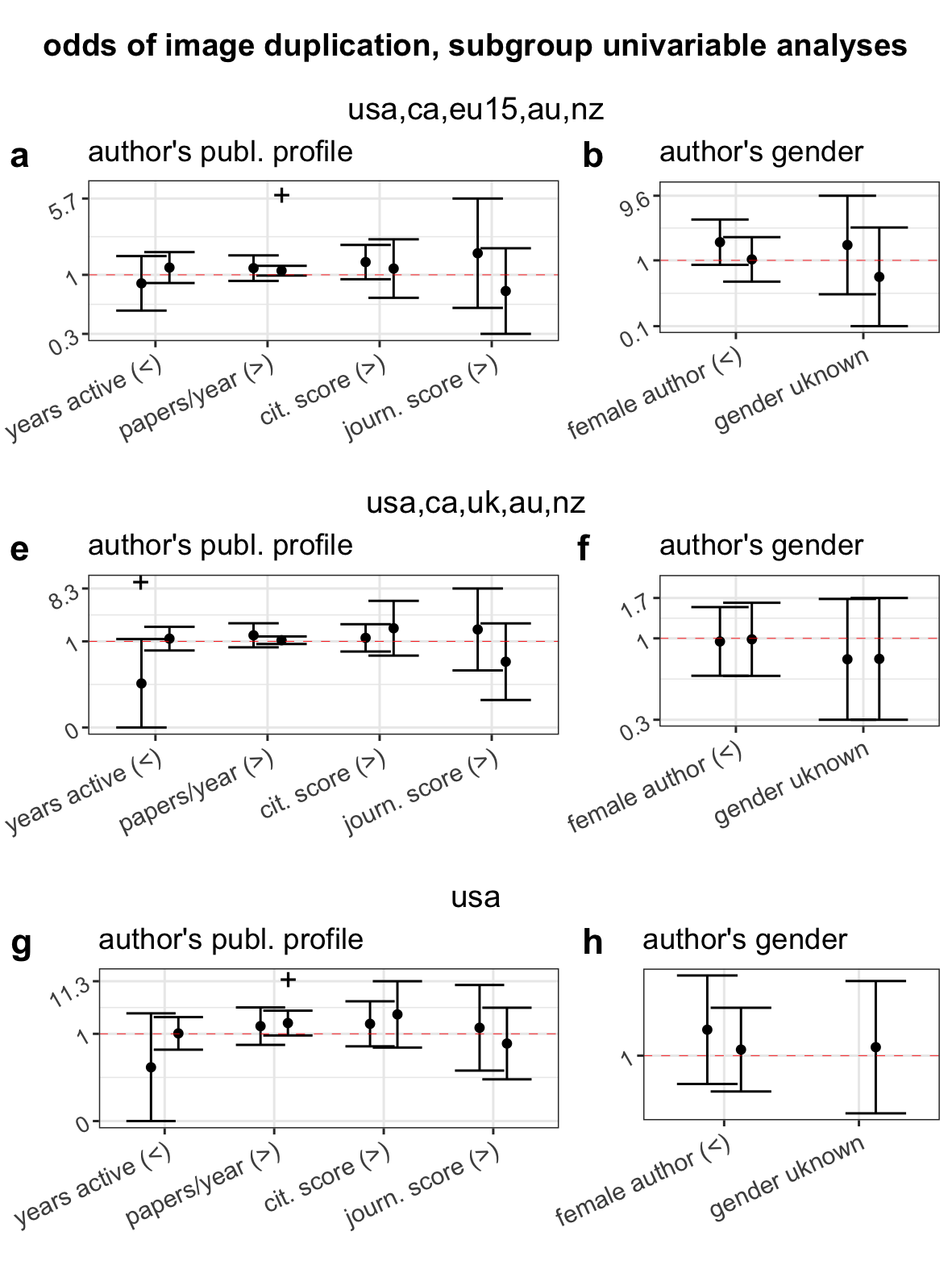

Supplement: Supplementary file 1 — Effect (Odds Ratio and 95% CI) of characteristics of first and last author on probability of publishing a paper containing a category 2 or 3 image duplication. Each subpanel shows results of univariable analyses on subsets of countries (see text for further details). First and second error bar correspond to data from first and last authors, respectively. Panels are subdivided according to overall hypothesis tested, and signs in parentheses indicate direction of expected effect (“>” : OR > 1; “<” : OR < 1). The more shifted the error bars are from the value of OR = 1 (dotted horizontal line), the larger the magnitude of effect measured. Bars that do not overlap with the OR = 1 line indicate an effect that is statistically significant at the 0.05 level or lower. Conventional thresholds of statistical significance are flagged above each error bar to facilitate effect estimation (“+”: p < 0.1; “*”: P < 0.05; “**”: P < 0.01; “***”: P < 0.001) (TIFF 7264 kb) [file 11948_2018_23_MOESM1_ESM.tiff]
